# Supplementary material for: Marker-Assisted Hybridization and Selection for Fiber Quality Improvement in Naturally Colored Cotton (G. hirsutum L.)
Source: Plants (Basel). 2025 Nov 26;14(23):3601. doi: 10.3390/plants14233601 (PMC12694247; doi:10.3390/plants14233601)
Supplement: Supplementary file 1 [file plants-14-03601-s001.zip › Table S2.docx]

**Table S2.** Cotton samples with white and colored fiber involved in the research.

| **No.** | **Samples** | **Origin** | **Source**  **(Germplasm collection)** | **Fiber character**  **(color)** |
| --- | --- | --- | --- | --- |
| 1. | cv. C-6580 | Uzbekistan | CBSPARI | White |
| 2. | cv. C-6570 | Uzbekistan | CBSPARI | White |
| 3. | cv. C-6577 | Uzbekistan | CBSPARI | White |
| **4.** | **L-**4068 | Uzbekistan | CBSPARI | Green |
| **5.** | **L-**4083 | Uzbekistan | CBSPARI | Brown |
| **6.** | **L-**4017 | Uzbekistan | CBSPARI | Brown |
| **7.** | **L-**4099 | Uzbekistan | CBSPARI | Light brown |
| **8.** | **L-**4092 | Uzbekistan | CBSPARI | Light brown |
| 9. | F3C-6570x4068 | Uzbekistan | CBSPARI | Green |
| 10. | F3C-6577x4068 | Uzbekistan | CBSPARI | Green |
| 11. | F3C-6580x4068 | Uzbekistan | CBSPARI | Green |
| 12. | F3C-6570x4083 | Uzbekistan | CBSPARI | Brown |
| 13. | F3C-6570x4017 | Uzbekistan | CBSPARI | Brown |
| 14. | F3C-6570x4099 | Uzbekistan | CBSPARI | Light brown |
| 15. | F3C-6570x4092 | Uzbekistan | CBSPARI | Light brown |
| 16. | F3C-6577x4083 | Uzbekistan | CBSPARI | Brown |
| 17. | F3C-6577x4017 | Uzbekistan | CBSPARI | Brown |
| 18. | F3C-6577x4092 | Uzbekistan | CBSPARI | Light brown |
| 19. | F3C-6577x4099 | Uzbekistan | CBSPARI | Light brown |
| 20. | F3C-6580x4083 | Uzbekistan | CBSPARI | Brown |
| 21. | F3C-6580x4017 | Uzbekistan | CBSPARI | Brown |
| 22. | F3C-6580x4099 | Uzbekistan | CBSPARI | Light brown |
| 23. | F3C-6580x4092 | Uzbekistan | CBSPARI | Light brown |
