# Supplementary material for: Marker-Assisted Hybridization and Selection for Fiber Quality Improvement in Naturally Colored Cotton (G. hirsutum L.)
Source: Plants (Basel). 2025 Nov 26;14(23):3601. doi: 10.3390/plants14233601 (PMC12694247; doi:10.3390/plants14233601)
Supplement: Supplementary file 1 [file plants-14-03601-s001.zip › Table S1.docx]

**Table S1.** SSR markers linked to fiber quality traits in cotton (Gossypium hirsutum L.), with primer sequences, map positions, associated traits, and predicted in silico PCR product sizes.

| **№** | **SSR marker** | **Primer sequences** | **Map** | **Chr.** | **cM** | **Chr. Position (Upstream/Downstream)** | ***In silico* PCR product size** |
| --- | --- | --- | --- | --- | --- | --- | --- |
| 1 | NAU4030 | TTGCTGTCATGGGTCATATC  CAAAACAACGGTGGTGTAAG | Guo *et al.* 2008 | A07/D07 | 13/19.3 | 2610819/2610973 2522926/2523089 | 155/164 |
|  |  |  |  |  |  |  |  |
| 2 | NAU5152 | TCCTTTCTACCCATGCCTAC  GTCACGAGAAGCAGAGGACT | Guo *et al.* 2008 | D07 | 34.4 | 4590871/4591095 | 225 |
|  |  |  | Blenda *et al.* 2012 | c7 | 127 |  |  |
| 3 | NAU2432 | AACTACAAAAGGATGAACATGG  TAGGGTTATTGCAGGCTAGG | Guo *et al.* 2008 | A07/D07 | 24.1/70.7 | 22558857/22559009 17598257/17598414 | 153/158 |
|  |  |  |  |  |  |  |  |
| 4 | NAU2186 | CAAAACGCTTTCGAATACAA  GATTACACCGCAGAGTCCTT | Guo *et al.* 2008 | A07 | 25.5 | 16769985/16770144 | 160 |
|  |  |  | Blenda *et al.* 2012 | с7 | 49 |  |  |
| 5 | NAU2887 | CACCATGAGCCACTAATTCA  ACACATTTTTCCCTTTTTGG | Guo *et al.* 2008 | D07 | 60.9 | 11884211/11884638 | 428 |
|  |  |  |  |  |  |  |  |
| 6 | NAU3180 | AGCAATGAAACCATAAACTG  GTGCTCCGGTAAATTTCATT | Guo *et al.* 2008 | D07 | 81.0 | 47803242/47803466 | 225 |
|  |  |  | Blenda *et al.* 2012 | с7 | 68 |  |  |
| 7 | BNL1694 | CGTTTGTTTTCGTGTAACAGG  TGGTGGATTCACATCCAAAG | Guo *et al.* 2008 | A07/D07 | 68.4/78.8 | 40367154/40367393 27936319/27936542 | 240/224 |
|  |  |  | Yu *et al.* 2014 | A07 | 52.3 |  |  |
|  |  |  | Blenda *et al.* 2012 | с7 | 65/68 |  |  |
| 8 | NAU1222 | ATAGGCCCATGTTGGAATTA  GTAGCCAGAAGTCCATGGTT | Guo *et al.* 2008 | A07 | 67.5 | 41307964/41308189 | 226 |
|  |  |  |  |  |  |  |  |
| 9 | NAU2995 | CGCTTTTATGCATGTAATCCT  CACAAGCTACAATTCCACCA | Guo *et al.* 2008 | D07 | 70.1 | 24883275/24883472 | 198 |
|  |  |  | Blenda *et al.* 2012 | с7 | 63 |  |  |
| 10 | BNL1604 | AGAGGGAGTAAAGATTTGGGG  TCCAGTTCTTTTTGCCTTGG | Guo *et al.* 2008 | A07/D07 | 73.0/92.4 | 91031923/91032024  52068992/52069112 | 102/121 |
|  |  |  | Yu *et al.* 2014 | A07/D07 | 31.2/46.0 |  |  |
|  |  |  | Blenda *et al.* 2012 | с7/с16 | 60/36 |  |  |
| 11 | BNL1122 | TCGATAACGGCTATAGTAATCTCTC  CAACAAATAAGCAGCCAAGAAA | Guo *et al.* 2008 | D07 | 87.8 | 45447159/45447330 | 172 |
|  |  |  | Yu *et al.* 2014 | A07/D07 | 53.5/55.2 |  |  |

**Note:** Marker positions are based on previously published genetic maps (Guo et al., 2008; Blenda et al., 2012; Yu et al., 2014) and aligned with the G. hirsutum reference genome (Texas RSI NG 8 assembly; NCBI Taxon ID: 3635).
